# Supplementary material for: Open-source microscope add-on for structured illumination microscopy
Source: Nat Commun. 2024 Feb 20;15:1550. doi: 10.1038/s41467-024-45567-7 (PMC10879112; doi:10.1038/s41467-024-45567-7)
Supplement: Supplementary file 1 — Supplementary information [file 41467_2024_45567_MOESM1_ESM.pdf]

Open-source microscope add-on for structured illumination microscopy

Mélanie T.M Hannebelle* [1,2,3], Esther Raeth* [1,2], Samuel M. Leitao[1], Tomáš Lukeš[1], Jakub Pospíšil[4,5], Chiara Toniolo[2], Olivier F. Venzin[2], Antonius Chrisnandy[2], Prabhu Prasad Swain[1], Nathan Ronceray[1], Matthias P. Lütolf [2], Andrew C. Oates[2], Guy M. Hagen[6], Theo Lasser[1], Aleksandra Radenovic[1], John D. McKinney[2], Georg E. Fantner† [1]

* These authors contributed equally to this work

†Corresponding author: georg.fantner@epfl.ch

[1] School of Engineering, Swiss Federal Institute of Technology (EPFL), Lausanne, Switzerland
[2] School of Life Sciences, Swiss Federal Institute of Technology (EPFL), Lausanne, Switzerland
[3] Center for Innovation in Global Health, Stanford University, Stanford, California, US
[4] Faculty of Electrical Engineering, Czech Technical University in Prague, Prague, Czech Republic
[5] Department of Medical Biology, UiT The Arctic University of Norway, Tromsø, Norway
[6] BioFrontiers Center, University of Colorado Colorado Springs, Colorado, US

Supplementary figures

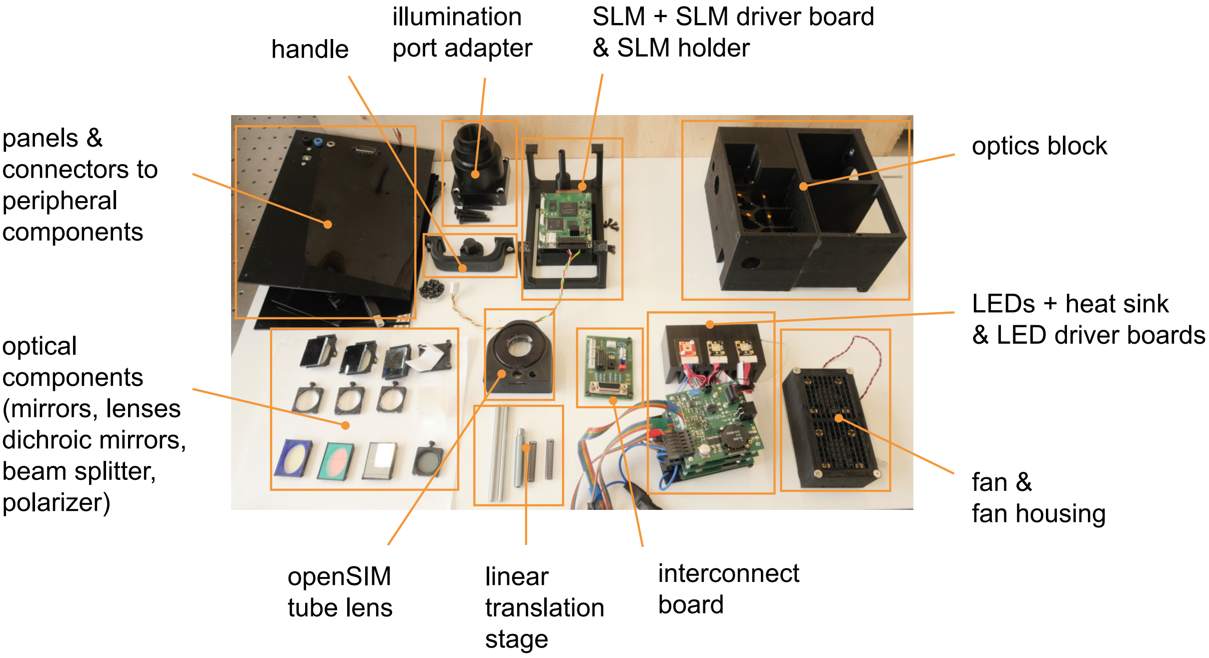

Supplementary Figure 1 Components of the openSIM.

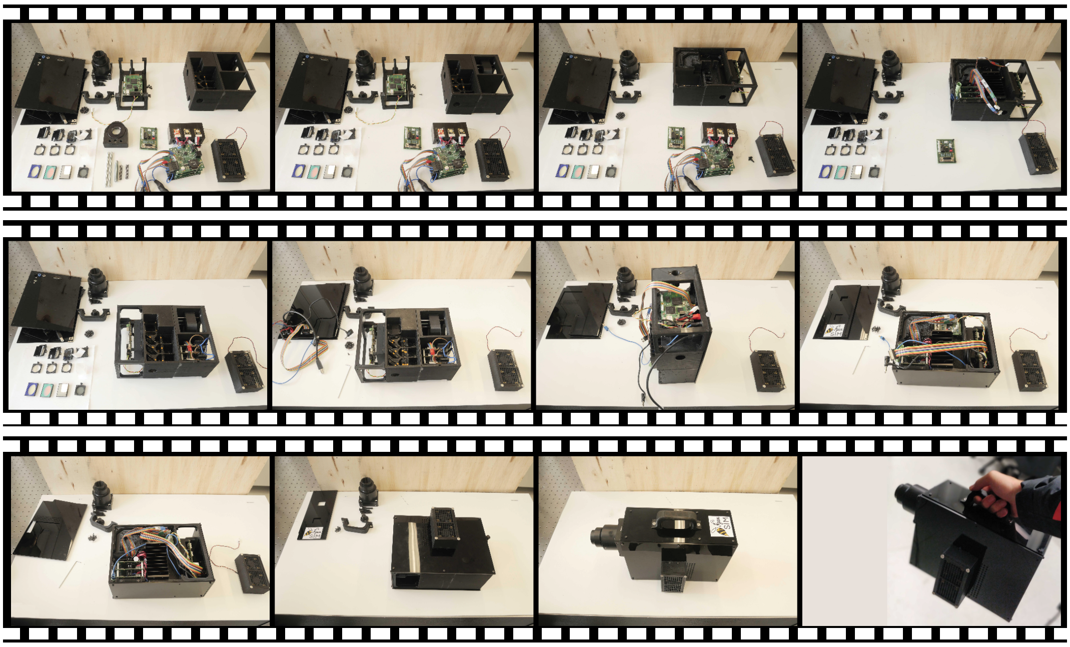

Supplementary Figure 2 Time-lapse of the assembly of an openSIM.

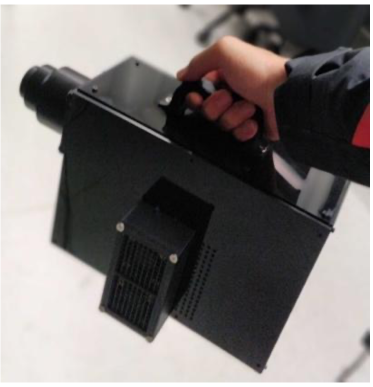

Supplementary Figure 3 Portable design of openSIM.

The openSIM is portable and can be moved easily from one instrument to another.

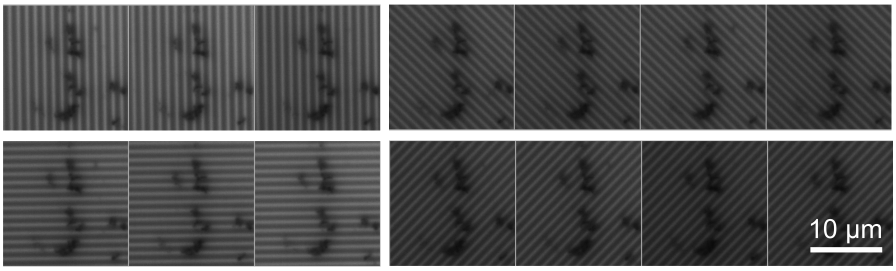

Supplementary Figure 4 Patterned illumination with the openSIM

Patterned illumination with the openSIM on a thin fluorescent film.

In this example, 4 angles are used and 14 different patterns.

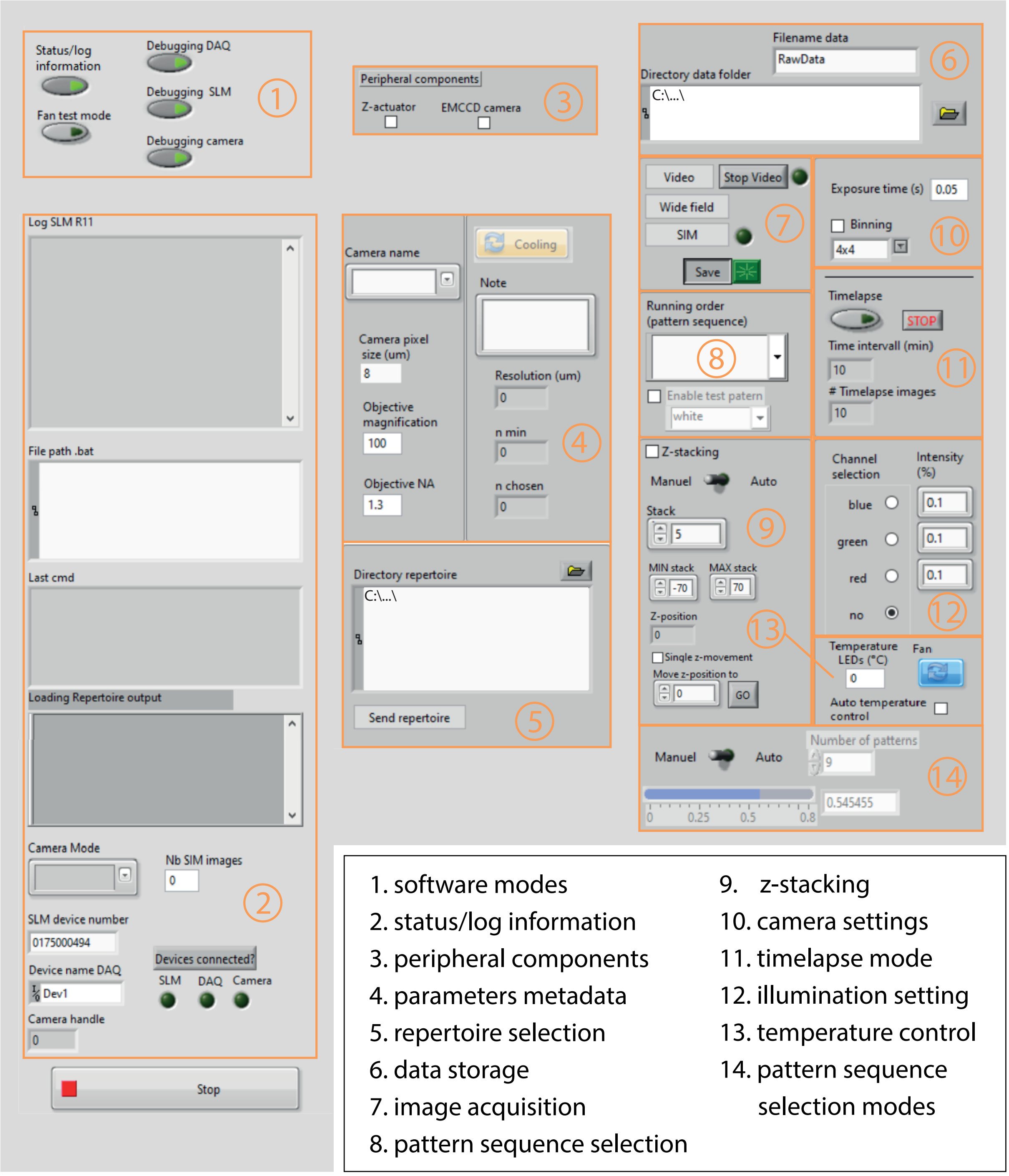

Supplementary Figure 5 User interface of the openSIM LabView software.

The openSIM software integrates the various components of openSIM including SLM, DAQ and camera, enabling efficient control over pattern generation, illumination management, temperature regulation, and data handling. Its modular architecture enables users with the flexibility to customize and tailor the software to their specific needs and requirements.

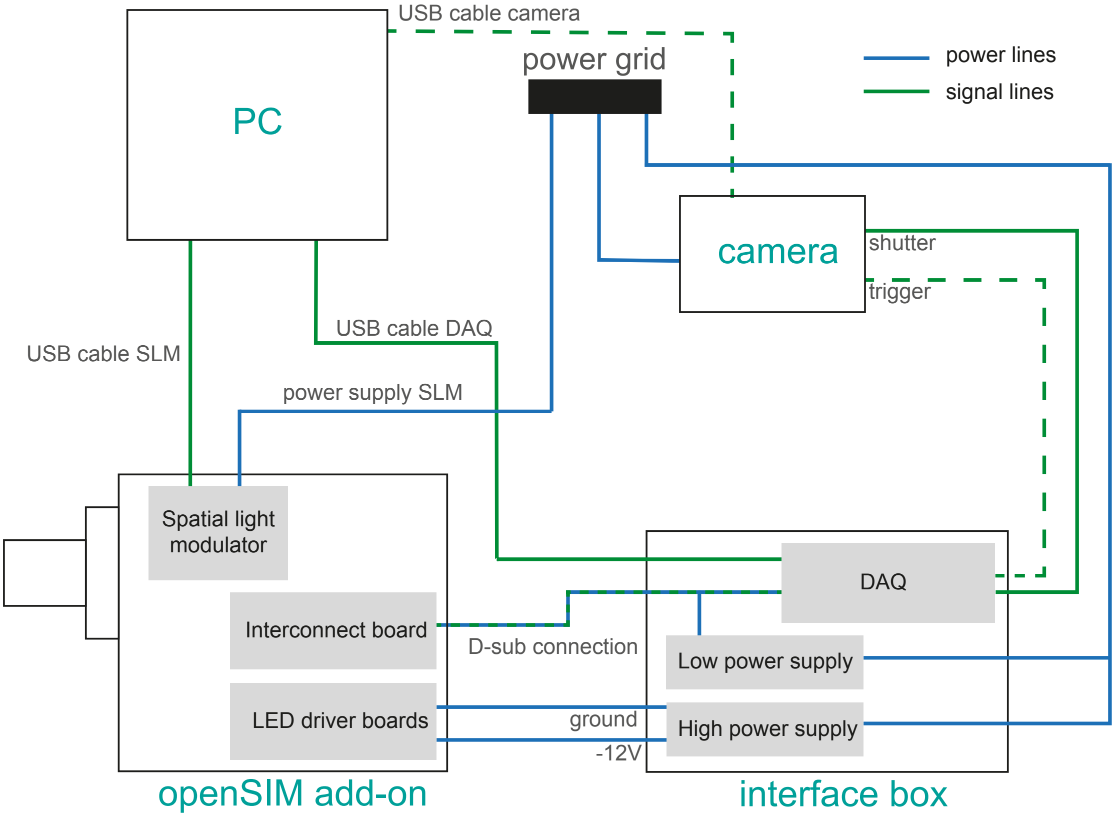

Supplementary Figure 6 Schematic of component connections.

Schematic of the connections between the different components of the openSIM system.

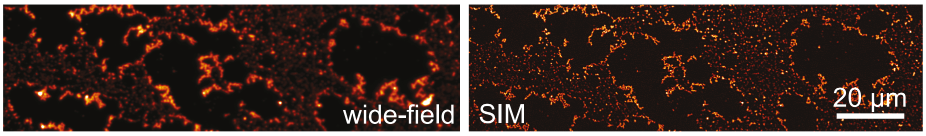

Supplementary Figure 7 Comparison between SIM and wide-field images of fluorescent beads with openSIM.

Comparison between an image of 100 nm beads with wide-field blue illumination compared to the openSIM image.

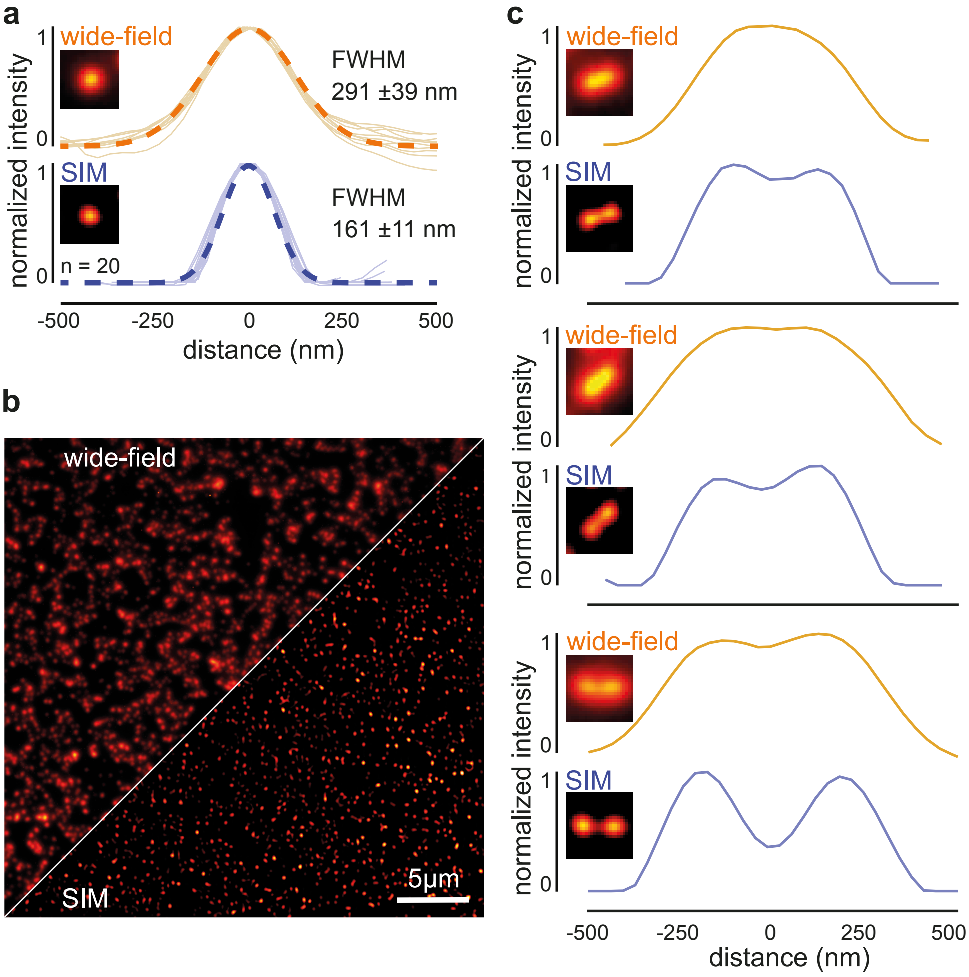

Supplementary Figure 8 Comparison between SIM and wide-field images of fluorescent beads with openSIM.

a Comparison between the point spread function of isolated 100 nm beads (TetraSpeck beads, 100 nm diameter), with wide-field illumination (top) and with the openSIM (bottom), with blue illumination. FWHM: full width at half maximum. Dotted line: average Gaussian fit. The picture inset is a representative point spread function. **b** Comparison between an image of 100 nm beads with wide-field blue illumination compared to the openSIM image. **c** Comparison of the resolving performance between the openSIM and wide-field illumination of two individual 100 nm beads placed close to each other.

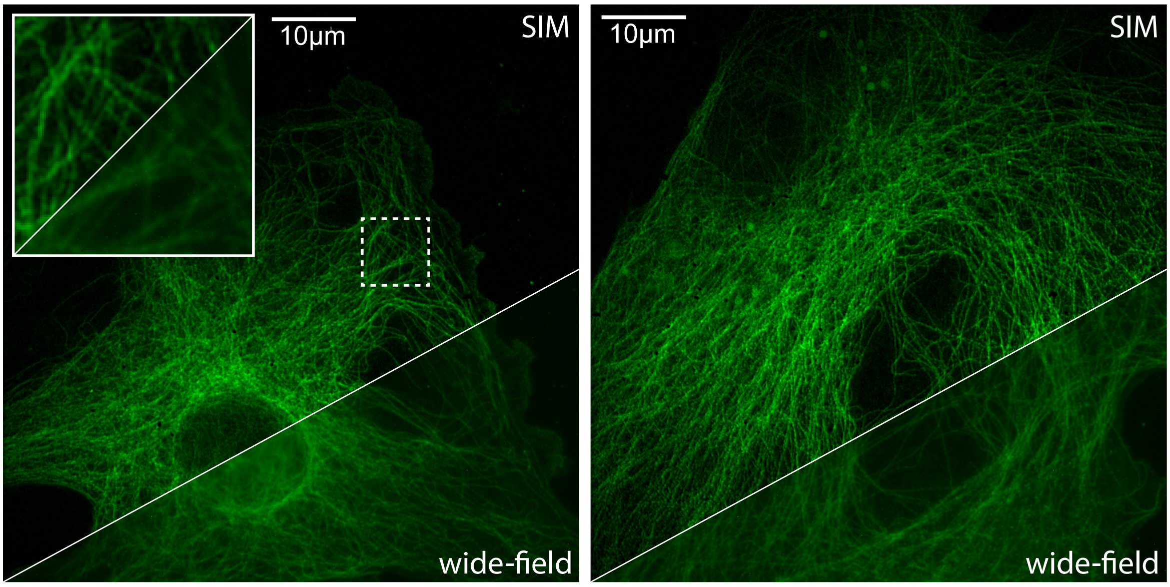

Supplementary Figure 9 Wide-field and SIM images of microtubules with openSIM.

Images of fixed bovine pulmonary artery endothelial cells, α-tubulin labeled with BODIPY. The inset represents a zoom in the area highlighted with a dotted square.

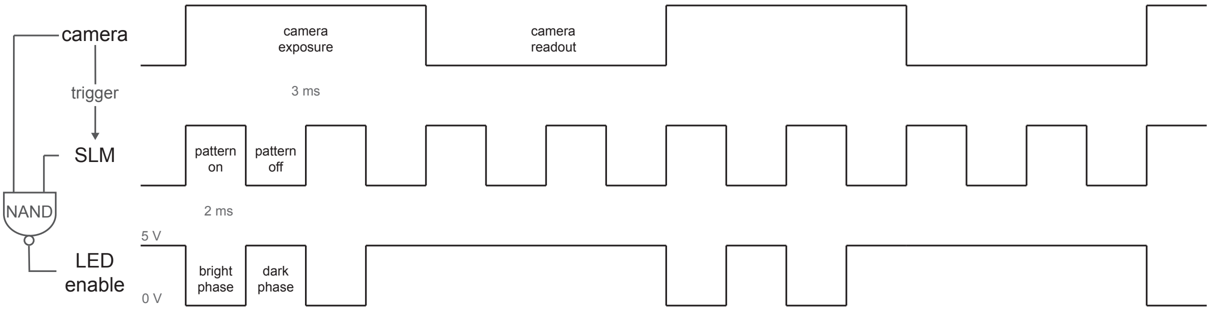

Supplementary Figure 10 Timing schedule to synchronize camera, SLM and LED.

Diagram showing the timing schedule of camera, SLM and LED and their interdependency. A possible example timing scheme is indicated underneath in grey. The rising and falling edge of the camera exposure signal triggers the progressive projection of the patterns of the selected pattern sequence. In order to ensure refresh cycles for the SLM, the LED is synchronized to the SLM and is turned off during the period in which the pattern is reversed. Important note: 0V of LED enable corresponds to LED on and 5V of LED enable to LED off respectively.

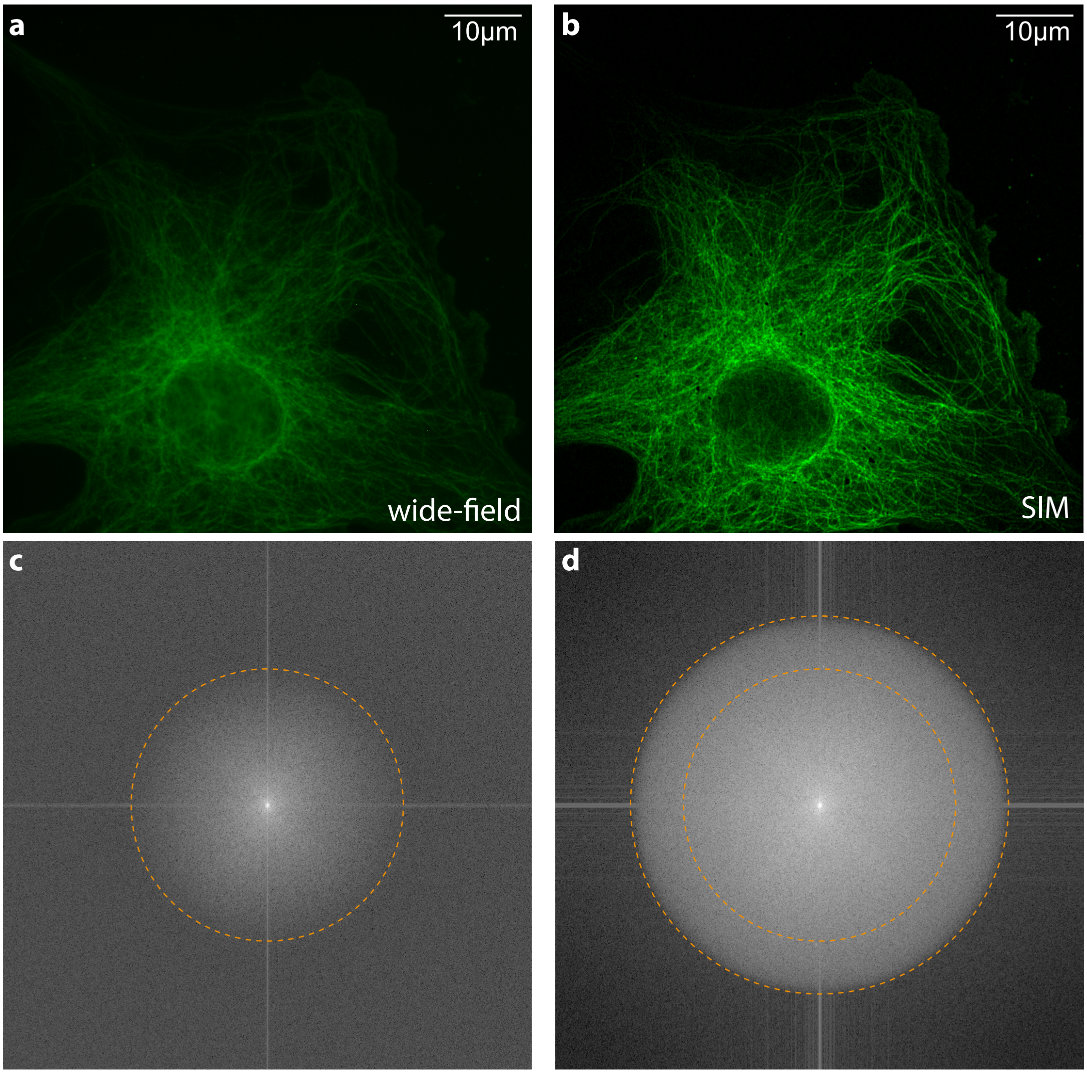

Supplementary Figure 11 Comparison between the Fourier transforms of widefield and SIM images.

Images of fixed bovine pulmonary artery endothelial cells, α-tubulin labeled with BODIPY and corresponding FFTs: **a** widefield image **b** SIM image **c** FFT of widefield image, the dotted circular line (~250 nm) indicates the conventional resolution limit **d** FFT of SIM. The dotted inner circular line (~250 nm) indicates the conventional resolution limit and the outer circular line (~180 nm) the approximate achieved resolution for this image. A Wiener constant of 1 was used during the SIM image reconstruction.
